# Comorbidity in patients with Lichen sclerosus: a retrospective cohort study

**DOI:** 10.1186/s40001-023-01335-9

**Published:** 2023-09-11

**Authors:** Sandra Jerkovic Gulin, Filippa Lundin, Oliver Seifert

**Affiliations:** 1 Department of Dermatology and Venereology, Ryhov County Hospital, Sjukhusgatan, 553 05 Jönköping, Sweden; 2 Division of Cell Biology, Department of Biomedical and Clinical Sciences, The Faculty of Medicine and Health Sciences, Linköping University, 581 83 Linköping, Sweden; 3 The Faculty of Medicine and Health Sciences, Linkoping University, 581 83 Linköping, Sweden

**Keywords:** Lichen sclerosus, Vulva cancer, Breast cancer, Penis cancer, Comorbidity

## Abstract

Lichen sclerosus (LS) is a chronic lymphocyte mediated inflammatory mucocutaneous disease of unknown aetiology with a predilection for the anogenital region, and affecting both sexes. The disease is characterized by pain, intolerable itching and scarring. In late stages of LS, disfiguring scarring can drastically alter the structural anatomical architecture of the genitals. The association between genital LS and different malignant tumours is a concern that needs to be further investigated. An association between LS and several autoimmune diseases has been confirmed in recent studies. All registered citizens of Region Jönköping, Sweden were included in the present study. Patients diagnosed with LS (*n* = 5680) between 2001 and 2021 were identified using ICD-10 code L90.0 and selected as cases. All other individuals (*n* = 362 568) served as controls. Odds ratios (ORs) for the selected comorbidity were calculated and adjusted for age and sex. The cumulative incidence of LS for the entire population over a 20-year period was 1.54% (15.4 per 1000 people). The cumulative incidences over a 20-year period for females and males were 2.13% and 0.97%, respectively. This study confirmed the association between LS and vulvar cancer (OR = 17.4; 95% CI 12.1–25.3), penis cancer (OR = 9.1; 95% CI 4.3–18.9), prostate cancer (OR = 2.0; 95% CI 1.6–2.4) and breast cancer (OR = 1.6; 95% CI 1.4–1.8). LS was also associated with Crohn´s disease (OR = 2.0; 95% CI 1.6–2.6) and diabetes mellitus type 1 (OR = 1.9; 95% CI 1.6–2.1). The present study revealed novel important data regarding the association of LS with cancer and autoimmune diseases, emphasising the importance of sufficient treatment and follow-up of patients with LS. However, future studies are needed to confirm these results and the potential role of LS in the development of cancer.

## Introduction

Lichen sclerosus (LS) is a chronic lymphocyte mediated inflammatory mucocutaneous disease with a predilection for the anogenital region [1]. The disease is characterized by intolerable itching, pain as a result of erosions or fissures, and scarring [2]. Untreated, LS can lead to the advanced stage of the disease with drastic alterations of the structural anatomical architecture in both sexes, ulcerative lesions with hemorrhage on the vulva, a loss of the labia minora, scarring and narrowing of the vaginal introitus, urine retention, anal stenosis, constipation [3] and phimosis. As a result, patients often experience both sexual and urinary dysfunction and might need reconstructive surgery. The first-line treatment consists of a potent topical steroid (clobetasol ointment) applied to the affected area. Circumcision is a treatment option in male patients that do not respond to topical steroids, and might be curative. If the diagnosis is delayed or the patient is unresponsive to steroids, genital reconstruction might be needed to regain function [4]. If left untreated, the vulvar area affected by LS can transform into a premalignant lesion or vulvar squamous cell carcinoma (SCC). Penile LS could also lead to penile SCC [2, 5].

Neither the pathophysiological mechanism, nor the etiology of the disease is known but theories suggesting autoimmunity, isotraumatopic or infectious geneses have been presented. Circulating IgG autoantibodies to the glycoprotein extracellular matrix protein 1 (ECM1) have been found in both sexes [6]. Associations between penile LS and human leukocyte antigens (HLA) class II have been found; however, similar HLAs appeared to be protective in vulvar LS [7]. Possible hypotheses for the development of LS include chronic exposure to trapped urine, supported by the fact that circumcision is often curative in the early stages of the disease [4]. Extragenital LS has in some cases appeared in the area of a surgery wounds [8], radiation therapy [9] and previous sunburns [10], suggesting that trauma might predispose the subject to LS. Previous studies have found that 12% of patients with LS report cases of LS in their family history and propose a genetic background to the development of the disease [7]. Further studies are required to fully understand the etiology of LS.

All age groups can be affected but overall females seem to be more prone than males to develop LS [2]. In vulvar LS there are two peak ages of presentation, in the prepubertal and postmenopausal years [3]. Similarly, there is also a bimodal onset of LS in males, with age peaks in young boys and adult men [11]. Due to the lack of large epidemiological studies, it is not possible to determine the true incidence of LS [2, 12]. LS has an estimated prevalence of 1:60 to 1:1,000 in adults and children in the United States [13] but since LS can be asymptomatic and is an under-recognized disease, these prevalences are probably underestimated [11]. A Dutch study found that the incidence of vulvar LS had increased from 7.4 per 100,000 woman-years in 1991 to 14.6 per 100,000 woman-years in 2011. The same study has found the mean age at the time of vulvar LS diagnosis was 59.8 years and the cumulative vulvar SCC incidence was 6.7% [14]. Another study found that the lifetime risk of penile SCC was 4–5%. The incidence of LS in penile cancer is relatively high [4, 15]. No epidemiological studies on LS have been conducted in Sweden.

The association between LS and vulvar SCC has previously been studied and is well established [2]; however, there are only a few studies investigating whether an association exists between LS and other types of cancer. The association between LS and thyroid disease has been established in women, but is not clear in men [2]. Several case reports and smaller studies have found an association between psoriasis [16], vitiligo [17], lichen planus [18], alopecia areata and ulcerative colitis [19, 20].

Furthermore, the coexistence of LS and morphea have sporadically been described in literature; however, a possible relationship between the diseases has been debated [21]. The comorbidity in patients with LS has not been analyzed in a Swedish population.

Today´s knowledge about LS is limited among medical professionals, sometimes resulting in wrong or delayed diagnosis, and inadequate treatment and follow-up. The lack of knowledge among the general population, as well as the hesitation to seek medical attention for genital symptoms, results in delayed diagnosis and treatment, severe genital disease with scarring, persistent urogenital dysfunction and, in some cases, cancer. This also impedes the effort to acquire a better understanding of the disease.

The primary aim of this study is to investigate whether the selected diagnoses are overrepresented in patients with LS compared to controls without LS. Identifying comorbidity that affects patients with diagnosed LS might help us to understand the, as of now, unknown pathogenesis of LS.

The secondary aim is to spread awareness about LS and its potential risk of developing into cancer, and to encourage further studies of LS to understand its etiology, pathophysiology and malignant potential.

## Methods

In this retrospective cohort study, all registered citizens of Region Jönköping, Sweden were included. Patients diagnosed with LS (ICD-10 code L90.0) in Cosmic R8, the journal software used by the health care provider, between 2001-01-01 and 2021-01-01 were identified and selected as cases. The control group includes all remaining registered citizens of the region at the start of the study (data obtained 2022-06-17). Likewise, Cosmic R8 was used to extract data on the prevalence of 28 other diagnoses (Table 2). The diagnoses were selected based on previous studies, our clinical experience and patient history reports. The age of the patients in the case group is described as the age when they were first diagnosed with LS, and the age of the controls is described as current age at 2020-12-31. The prevalence of the selected diagnoses in patients with LS was compared to the prevalence of the same diseases in the control group.

### Ethical considerations

This registry-based study utilizes strictly pseudonymised data and abides by the General Data Protection Regulation; therefore, no consent from the subjects was needed. Ethical approval from the Swedish Ethical Review Authority was obtained 2021-11-15: dnr. 2021-05590-01.

### Statistical analysis

The data collected from Cosmic R8 were compiled into an anonymized file, except for sex, age and disease code, to be used in IBM SPSS version 28.0 for analysis.

The cumulative incidence of developing LS was analyzed. The odds ratio (OR) and adjusted OR, 95% confidence intervals (95% CIs), as well as *p*-values for each diagnosis were calculated using binominal logistic regression. All ORs were adjusted for age and in the analysis of diagnoses which can occur in both women and men, the ORs were also adjusted for sex. *P* < 0.05 was considered statistically significant.

The linearity of the continuous variable ´age´ with respect to the logit of the dependent variable was assessed via the Box-Tidwell procedure. Based on this assessment, the continuous independent variable age was found to be not linearly related to the logit of the dependent variable. Hence, we transformed the variable ´age´ into age group strata to avoid violation of the linearity assumption in logistic regression.

## Results

**Fig. 1:**
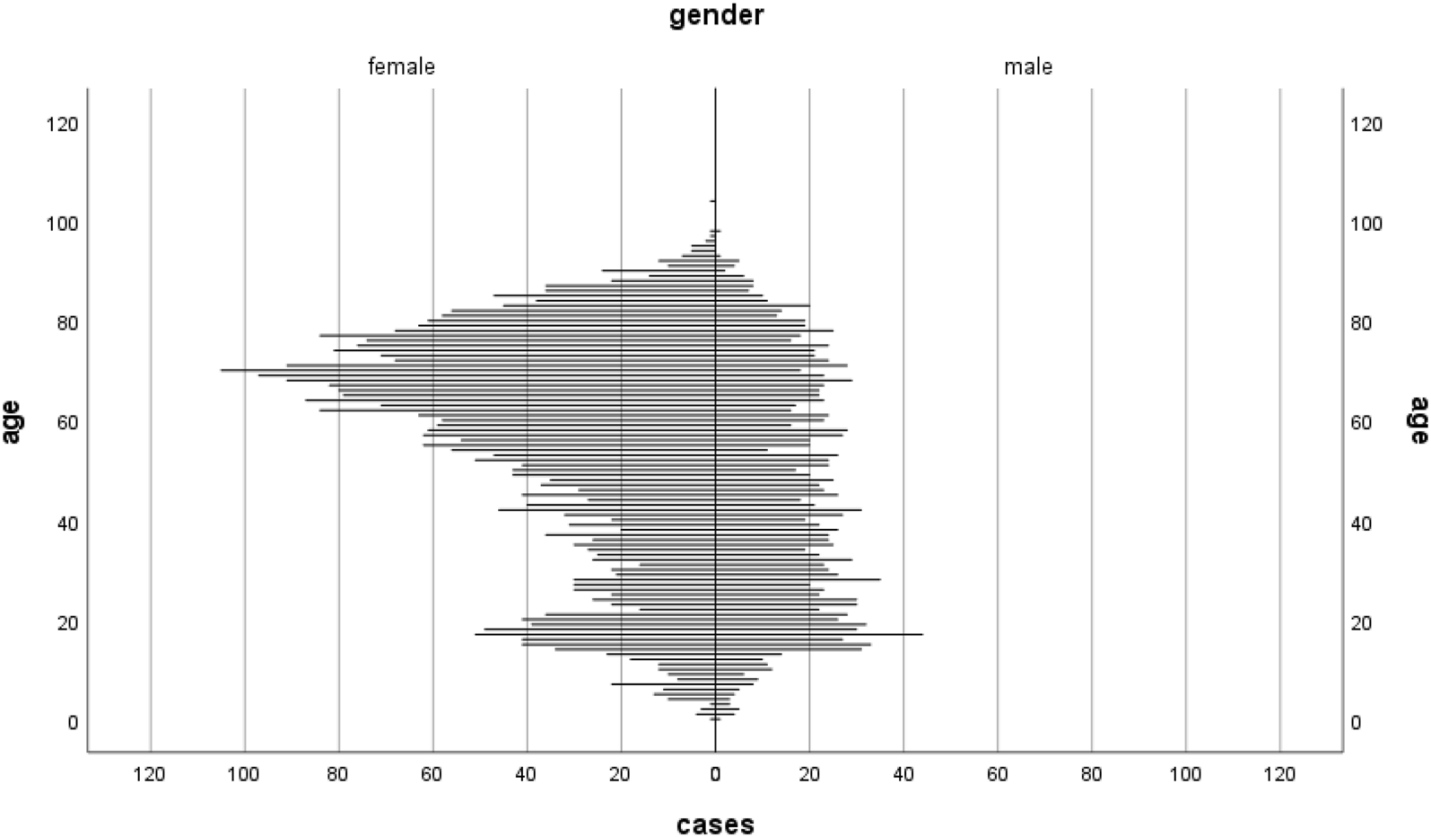
Population pyramid depicting the age dispersion of patients with lichen sclerosus, split by sex (females *n* = 3878, males *n* = 1802)

A total of 368,248 people were included in this study. Among these, 5680 (1.5%) were included in the case group and 362,568 (98.5%) in the control group. 1802 males and 3,878 females were included in the case group and 184,848 males and 177,720 females in the control group. The median age in the case group was 57 years and the highest percentage of patients, 18.6%, were in the age group 61–70 (Fig. 1).

The median age of the control group was 40 years (Table 1). The cumulative incidence for the entire population over a 20-year period was 1.54% (15.4 per 1000 people). The cumulative incidences for females and males over a 20-year period were 2.13% and 0.97% respectively. The results indicated that patients with LS had an increased risk of several of the malignant diagnoses and premalignant lesions studied, including vulvar cancer (OR = 17.4; 95% CI 12.1–25.3), penis cancer (OR = 9.1; 95% CI 4.3–18.9), prostate cancer (OR = 2.0; 95% CI 1.6–2.4), breast cancer (OR = 1.6; 95% CI 1.4–1.8), leukoplakia of vulva (OR = 361.1; 95% CI 274.8–474.6) and leukoplakia of penis (OR = 3.3; 95% CI 2.9–3.7) (Table 2).

**Table 1:** Table showing demographics of patients with lichen sclerosus and controls

	Lichen Sclerosus				Controls			
	*n* 5680	% 1.5	M 1802	F 3878	*n* 362,568	% 98.5	M 184848	F 177720
Age								
Median (min–max)	57 (0–104)		44 (0–98)	61 (0–104)	40 (0–110)		39 (0–104)	41 (0–110)
0–10	155	2.7	60	95	49,336	13.6	25,290	24,046
11–20	607	10.7	258	349	43,212	11.9	22,470	20,742
21–30	515	9.1	260	255	46,574	12.8	24,903	21,671
31–40	492	8.7	233	259	44,849	12.4	23,291	21,558
41–50	603	10.6	230	373	43,656	12.0	22,360	21,296
51–60	770	13.6	219	551	44,740	12.3	23,080	21,660
61–70	1056	18.6	217	839	38,228	10.5	19,476	18,752
71–80	952	16.8	215	737	33,772	9.3	16,687	17,085
81 +	530	9.3	110	420	18,201	5.0	7291	10,910

*n* Number, *M* male, *F* female

**Table 2:** Number, frequency and odds ratio for comorbid conditions of patients with lichen sclerosus compared to controls

Comorbidity	ICD-10-code	Lichen sclerosus (n = 5680) n (%)	OR (95% CI)	Adjusted OR ^c^ (95% CI)	Significance level^d^
Leukoplakia of vulva^a^	N90.4	525 (9.2)	463.6 (354.2–606.9)	361.1 (274.8–474.6)	***
Leukoplakia of penis^b^	N48.0	351 (6.2)	3.3 (3.0–3.8)	3.3 (2.9–3.7)	***
Malignant tumor of penis^b^	C60	11 (0.2)	11.6 (5.6–24.1)	9.1 (4.3–18.9)	***
Malignant tumor of prostate^b^	C61	112 (2.0)	2.4 (2.0–2.9)	2.0 (1.6–2.4)	***
Malignant tumor of testicle^b^	C62	2 (0.0)	0.8 (0.2–3.1)	0.7 (0.2–2.9)	ns
Malignant tumor of urethra	C68	1 (0.0)	3.5 (0.5–26.6)	3.1 (0.4–23.5)	ns
Malignant tumor of vulva^a^	C51	47 (0.8)	29.1 (20.2–41.9)	17.4 (12.1–25.3)	***
Malignant tumor of breast	C50	228 (4.0)	3.7 (3.3–4.3)	1.6 (1.4–1.8)	***
Alopecia areata	L63	53 (0.9)	2.4 (1.8–3.2)	2.2 (1.6–2.9)	***
Ulcerative colitis	K51	89 (1.6)	2.1 (1.7–2.6)	1.8 (1.5–2.2)	***
Crohn´s disease	K50	62 (1.1)	2.4 (1.8–3.1)	2.0 (1.6–2.6)	***
Diabetes mellitus type 1	E10	208 (3.7)	2.4 (2.1–2.8)	1.9 (1.6–2.1)	***
Localized scleroderma (morphea)	L94	31 (0.5)	10.3 (7.0–15.0)	6.9 (4.7–10.2)	***
Systemic sclerosis	M34	11 (0.2)	7.1 (3.8–13.3)	4.2 (2.2–7.8)	***
Thyroiditis	E06	31 (0.5)	1.6 (1.1–2.2)	1.1 (0.7–1.5)	ns
Thyrotoxicosis (Grave´s disease)	E05	127 (2.2)	2.3 (1.9–2.8)	1.3 (1.1–1.6)	**
Myasthenia gravis	G70	6 (0.1)	2.7 (1.2–6.0)	1.8 (0.8–4.0)	ns
Multiple sclerosis	G35	13 (0.2)	1.1 (0.6–1.9)	0.7 (0.4–1.3)	ns
Guillain-Barré syndrome	G61	7 (0.1)	2.1 (1.0–4.4)	1.6 (0.7–3.4)	ns
Vitiligo	L80	52 (0.9)	3.0 (2.3–4.0)	2.8 (2.1–3.7)	***
Systemic lupus erythematosus	M32	17 (0.3)	3.5 (2.1–5.6)	2.1 (1.3–3.4)	**
Vasculitis	L95	34 (0.6)	5.7 (4.0–8.2)	3.9 (2.7–5.6)	***
Lichen ruber planus	L43	198 (3.5)	11.1 (9.5–12.9)	7.9 (6.8–9.2)	***
Lyme disease	A69.2	478 (8.4)	1.7 (1.6–1.9)	1.3 (1.2–1.4)	***
Seropositive rheumatoid arthritis	M05	56 (1.0)	2.2 (1.7–2.8)	1.2 (0.9–1.5)	ns
Other rheumatoid arthritis	M06	127 (2.2)	2.8 (2.3–3.3)	1.6 (1.3–1.9)	***
Polyarteritis nodosa	M30	5 (0.1)	3.3 (1.3–8.1)	3.1 (1.2–7.6)	*
Other necrotizing vasculopathies	M31	37 (0.7)	3.2 (2.3–4.5)	1.9 (1.3–2.6)	***

^a^Only females were included in regression analysis

^b^Only males were included in regression analysis

^c^Odds ratio adjusted for age group and sex, if both sexes were included in regression analysis

^d^Significance level of adjusted odds ratio

(* *p*<0.05; ** *p*<0.01; *** *p*<0.001; ns *p*>0.05)

Furthermore, the study showed a positive association between LS and autoimmune diseases such as alopecia areata (OR = 2.2; 95% CI 1.6–2.9), ulcerative colitis (OR = 1.8; 95% CI 1.5–2.2), Crohn´s disease (OR = 2.0; 95% CI 1.6–2.6), diabetes mellitus type 1 (OR = 1.9; 95% CI 1.6–2.1) vitiligo (OR = 2.8; 95% CI 2.1–3.7) and vasculitis (OR = 3.9; 95% CI 2.7–5.6). While there was no significant association with seropositive rheumatoid arthritis (RA), the results indicated an association with other, non-seropositive, RA (OR = 1.6; 95% CI 1.3–1.9) (Table 2). Increased odds ratios in patients with LS were also seen for morphea (OR = 6.9; 95% CI 4.7–10.2), systemic sclerosis (OR = 4.2; 95% CI 2.2–7.8), systemic lupus erythematosus (OR = 2.1; 95% CI 1.3–3.4) and thyrotoxicosis of both autoimmune and other geneses (OR = 1.3; 95% CI 1.1–1.6). An increased risk for infection with Lyme disease (OR = 1.3; 95% CI 1.2–1.4) was shown as well as for several diseases of unknown geneses including lichen ruber planus (OR = 7.9; 95% CI 6.8–9.2), polyarteritis nodosa (OR = 3.1; 95% CI 1.2–7.6) and other necrotising vasculopathies (OR = 1.9; 95% CI 1.3–2.6) (Table 2). The current study did not find a significant association between LS and malignant tumors of the testicle or urethra, nor for thyroiditis, myasthenia gravis, multiple sclerosis or Guillain–Barré syndrome (Table 2).

## Discussion

### Bimodal disease onset only in female patients

The previously described bimodal age onset of LS in both sexes [11] could only be seen in the female population analyzed in this study. In males, only a definitive peak was shown in young men around the age of 20 years. We can only hypothesize about the reason for the missing peak of LS in older men. Our results might be explained by the fact that LS could present with only mild symptoms in older men, but they do not feel the need to seek medical attention and, therefore, will not get diagnosed. Another explanation is the missing age-dependent variations in serum estrogen in men compared to women leading to a continuous age distribution of LS in men [22]. Studies on postmenopausal women show that estrogen reduction leads to skin dryness, thinning and slow healing. These women also exhibit epidermal thinning, reduced collagen, lower skin moisture, decreased elasticity, and impaired wound healing [22]. The practice in Sweden does not encompass performing circumcision as a routine in newborn boys or upon request. Instead, it is exclusively carried out in cases of severe phimosis. An interesting aspect is that, in these instances, the excised tissue is frequently not subjected to histopathological examination, which could result in cases going undocumented. However, it's important to note that there is currently no available data on this specific aspect. As a result, we believe that its potential impact on our results is limited.

### LS poses a higher risk for malignancy

In accordance with previous studies, there was a strong association between LS and leukoplakia of the vulva as well as with vulvar cancer [2]. To our knowledge, there are, apart from some case reports, no larger studies showing an association between LS and penile cancer [23]. The current study shows that penile leukoplakia as well as penile tumors were more common in patients with LS, emphasizing the need for a nationwide study to confirm our data and evaluate the potential need for an update of the national guidelines for the diagnosis, treatment, screening and follow-ups of male patients with LS. A limitation inherent in our study pertains to the potential overlap between LS-diagnosed patients and those diagnosed with leukoplakia. This dual diagnosis could have an impact on our data, potentially leading to an overstatement of the association between LSc and leukoplakia. This consideration underscores the need for cautious interpretation of the observed link between these conditions in our findings.

Studies linking LS to higher or lower risks of other types of cancers than vulvar are scarce. The results in this study suggest that there is a positive association between LS and malignant tumors of the breast, which contradicts the results of a previous study [24]. It is difficult to explain these different results, but they may be due to genetic or geographical variations in the population of interest. To clarify these contradictory results, the relationship between LS and malignant tumors of the breast should be further investigated in larger nationwide register studies.

Furthermore, the current study revealed a positive association between LS and prostate cancer which has not been studied before. We included this malignancy in our study based on personal clinical observations and patient history that led to high clinical suspicion of positive associations between penile LS and prostate cancer.

There were no patients in this study with LS and concomitant malignant tumor of the urethra. In Sweden, urethral tumors are rare, and a nationwide study should be conducted to obtain valid results and to confirm, or reject, a possible association between the diseases. There was no significant association between LS and malignant tumors of the testicle, ureter, bladder or urethra. Larger nationwide studies are needed to investigate a possible association.

### Increased comorbidity in patients with LS

As in previous studies, there was no significant increase in seropositive RA among patients with LS compared to controls. However, the results showed an increased OR for other non-seropositive RA. There was a significant association between systemic lupus erythematosus (SLE) and LS in the current study. Concomitant SLE and LS have previously only been reported in a few case reports and more recently, an increased risk for SLE in LS patients was found in a Finnish case–control study [25]. An increased odds ratio was found for diabetes mellitus type 1 (DM1). A study conducted by Virgilli et al. in Italy has provided noteworthy insights. Their findings revealed that individuals affected by LS displayed a higher prevalence of being overweight or obese when compared to the broader Italian population [26]. An American study confirming older data suggested that autoimmune thyroid disease is more common in patients with LS with 2.67-, 2.88-, 2.34- and 2.05-fold increases in odds of having thyroiditis, autoimmune thyroiditis, hypothyroidism and hyperthyroidism, respectively [27]. The current study did not find a significant association between thyroiditis and LS despite the already established relationship in women [28]. However, this study shows that patients with LS have an increased risk for thyrotoxicosis, which is in line with previous studies [27]. Lichen ruber planus is commonly found coexisting with LS lesions [18]. The strong association presented in this study supports this.

The associations between LS and alopecia areata, ulcerative colitis [19] and vitiligo [17] shown in this study confirm previous findings of a relation of LS to these autoimmune diseases. However, this study has also shown an association between LS and Crohn´s disease, which has not been reported before.

Vasculitis has previously been reported to coexist with LS, possibly owing to the HLA-DR bearing keratinocytes demonstrated in both diseases and the consequent presence of lymphocytic infiltrate containing numerous activated T-cells [29].

The association between LS and Lyme disease shown in this study is in line with a study from 2008 in which focus-floating microscopy was used to detect *Borrelia burgdorferi *sensu lato, the spirochete that causes Lyme disease, in tissue specimens from patients with LS. Borrelia species were detected in 63% of LS patients, with detection being higher in the inflammatory phase of LS (80%) versus the atrophic phase (33.3%). The study has proposed *Borrelia burgdorferi *sensu lato as a trigger for LS. Some controversy remains regarding this association, primarily in the USA where it cannot be seen. The association has been identified in numerous studies involving European populations [30], yet its exact nature remains unclear [31].

Increased odds ratios in patients with LS were also seen for morphea. An American study observed a higher rate of LS in postmenopausal women with morphea, and all patients with genital involvement of morphea also had clinical and histologic features of LS. 59.2% of the patients with genital involvement had extragenital LS overlapping plaques of morphea. The coexistence of LS and morphea has been described previously, however, a possible correlation between the diseases has been debated [21]. A French study found a positive association between genital LS and limited cutaneous systemic sclerosis, a subset of systemic sclerosis (OR = 5.4; 95% CI 1.1–16.8). However, the study did not find an association between LS and systemic sclerosis including all subsets of the disease [32]. The current study did find a positive association between LS and systemic sclerosis, including all subsets, which motivates further studies on their suspected relationship.

### Strengths and limitations

There is potential bias in that some LS patients are probably missing from our cohort because not all affected patients receive the diagnosis due to mild symptoms, wrong diagnosis and patients not seeking help. Over the time the diagnostic criteria of LS have changed, possibly becoming more accurate. The diagnosis is mostly made on clinical grounds, not always confirmed by histopathology. We do not have data on whether the LS was confirmed by biopsy in the cases included in this study. Furthermore, ICD-10 codes are manually added to a patient´s journal, sometimes resulting in the wrong code being assigned by mistake. However, ICD-10 is an internationally recognized coding system for diagnoses for which health care workers have had training, and possible mistakes should be few, with limited significance for altering the results. Moreover, it's important to note that our case group is composed of individuals who are older than those in the control group. To mitigate the potential impact of this age difference on our findings, we took the necessary step of adjusting the results for age. This adjustment helps ensure that any observed associations are not solely attributed to age disparities between the two groups. This study showed that there are associations between LS and several other diagnoses that both confirm and contradict previous studies, and consequently that there is reason to further investigate LS. However, future studies should aim at garnering a bigger population with age- and sex-matched controls, which would generate a fairer picture of the risks associated with LS.

### Ethical aspects

Acquired data used for this study were pseudonymised, with each patient given only a number as an identifier. No sensitive data could, therefore, be traced back to an individual.

## Conclusion

The outcomes of this study not only validate the well-established risk of vulvar and penile cancer development but also uncover novel insights concerning LS. These newfound revelations affirm the clinical suspicions of a positive correlation between LS and malignant tumors of the prostate, breast, and other diseases. Nevertheless, to solidify the credibility of our findings, it becomes imperative to undertake a comprehensive nationwide study. Such an endeavor will not only reinforce the veracity of our data but also contribute significantly to the broader understanding of these intricate associations. The results will enable a better understanding of this, after all, not so rare disease, and it should lead to a different approach to treatment, cancer screening and follow-ups. Furthermore, the study raises new scientific questions which should result in further studies on the etiology and pathophysiology of the disease, with a main focus on the malignant potential of LS, which may lead us not only to a better understanding of the disease, but also to more effective new treatment options.

## Data Availability

Data and materials can be assessed by contacting one of the authors.
